# Adapting a home telemonitoring intervention for underserved Hispanic/Latino patients with type 2 diabetes: an acceptability and feasibility study

**DOI:** 10.1186/s12911-020-01346-0

**Published:** 2020-12-07

**Authors:** Renee Pekmezaris, Myia S. Williams, Briana Pascarelli, Kayla D. Finuf, Yael T. Harris, Alyson K. Myers, Tonya Taylor, Myriam Kline, Vidhi H. Patel, Lawrence M. Murray, Samy I. McFarlane, Karalyn Pappas, Martin L. Lesser, Amgad N. Makaryus, Sabrina Martinez, Andrjez Kozikowski, Jennifer Polo, Josephine Guzman, Roman Zeltser, Jose Marino, Maria Pena, Ralph J. DiClemente, Dilcia Granville

**Affiliations:** 1 Department of Medicine, Division of Health Services Research, Northwell Health, Manhasset, NY USA; 2 Donald and Barbara Zucker School of Medicine at Hofstra/Northwell, Hempstead, NY USA; 3 Feinstein Institute for Medical Research, Northwell Health, Manhasset, NY USA; 4 Center for Health Innovations and Outcomes Research, Northwell Health, Manhasset, NY USA; 5 Department of Medicine, Division of Endocrinology, North Shore University Hospital, Manhasset, NY USA; 6 College of Medicine, Division of Infectious Disease, SUNY-Downstate Health Sciences University, Brooklyn, NY USA; 7 Annie E. Casey Foundation Children and Family Fellowship, Baltimore, MD USA; 8 Department of Medicine, SUNY-Downstate Health Sciences University, Brooklyn, NY USA; 9 Department of Cardiology, Nassau University Medical Center, East Meadow, NY USA; 10 Northwell Health, Manhasset, NY USA; 11 Nassau University Medical Center, East Meadow, NY USA; 12 Mount Sinai Hospital, Mount Sinai Health System, New York, NY USA; 13 Department of Social and Behavioral Sciences, NYU School of Global Public Health, New York, NY USA; 14 Hispanic Counseling Center, Hempstead, NY USA

**Keywords:** Home telemedicine, Type 2 diabetes, Hispanic/Latino population, ADAPT-ITT, Feasibility

## Abstract

**Background:**

Home telemonitoring is a promising approach to optimizing outcomes for patients with Type 2 Diabetes; however, this care strategy has not been adapted for use with understudied and underserved Hispanic/Latinos (H/L) patients with Type 2 Diabetes.

**Methods:**

A formative, Community-Based Participatory Research approach was used to adapt a home telemonitoring intervention to facilitate acceptability and feasibility for vulnerable H/L patients. Utilizing the ADAPT-ITT framework, key stakeholders were engaged over an 8-month iterative process using a combination of strategies, including focus groups and structured interviews. Nine Community Advisory Board, Patient Advisory, and Provider Panel Committee focus group discussions were conducted, in English and Spanish, to garner stakeholder input before intervention implementation.

Focus groups and structured interviews were also conducted with 12 patients enrolled in a 1-month pilot study, to obtain feedback from patients in the home to further adapt the intervention. Focus groups and structured interviews were approximately 2 hours and 30 min, respectively. All focus groups and structured interviews were audio-recorded and professionally transcribed. Structural coding was used to mark responses to topical questions in the moderator and interview guides.

**Results:**

Two major themes emerged from qualitative analyses of Community Advisory Board/subcommittee focus group data. The first major theme involved intervention components to maximize acceptance/usability. Subthemes included tablet screens (e.g., privacy/identity concerns; enlarging font sizes; lighter tablet to facilitate portability); cultural incongruence (e.g., language translation/literacy, foods, actors “who look like me”); nursing staff (e.g., ensuring accessibility; appointment flexibility); and, educational videos (e.g., the importance of information repetition). A second major theme involved suggested changes to the randomized control trial study structure to maximize participation, including a major restructuring of the consenting process and changes designed to optimize recruitment strategies. Themes from pilot participant focus group/structured interviews were similar to those of the Community Advisory Board such as the need to address and simplify a burdensome consenting process, the importance of assuring privacy, and an accessible, culturally congruent nurse.

**Conclusions:**

These findings identify important adaptation recommendations from the stakeholder and potential user perspective that should be considered when implementing home telemonitoring for underserved patients with Type 2 Diabetes.

**Trial registration:**

NCT03960424; ClinicalTrials.gov (US National Institutes of Health). Registered 23 May 2019. Registered prior to data collection. https://www.clinicaltrials.gov/ct2/show/NCT03960424?term=NCT03960424&draw=2&rank=1

## Background

Over half of Hispanic/Latino (H/L) people will develop Type 2 Diabetes during their lifetime [1]. According to the Center for Disease Control, Type 2 Diabetes is a leading cause of suffering and death due to cardiovascular disease, end-stage renal failure, blindness, nontraumatic lower limb amputations, hospitalizations, and poor quality of life [2]. The risk of dying prematurely for people with Type 2 Diabetes is twice that of those without Type 2 Diabetes [1]. In the US, the prevalence in H/L males 65–74 years old is 31.1%; for H/L females in the same age group, it is 32.6% [3]. Compared to non-H/L whites, the risk of diagnosed Type 2 Diabetes is 66% higher among H/L patients [4]. The National Academy of Medicine reports that H/L patients experience a 50–100% higher illness burden and mortality from Type 2 Diabetes than non-H/L whites and that Type 2 Diabetes remains poorly managed [4]. H/L patients with Type 2 Diabetes have renal insufficiency rates 3 to 6 times greater than those of non-H/L white patients and end-stage renal disease rates are 41% higher [5–9]. For H/L patients, the prevalence of diabetic retinopathy is 84% higher than that of non-H/L whites [5, 7]. Compared to non-H/L whites, H/L Americans bear twice the risk of lower extremity amputation [9]. The literature unequivocally shows that racial/ethnic minorities with Type 2 Diabetes have poor access to healthcare and lower levels of Type 2 Diabetes management [10–13].

Home telemonitoring is a promising approach to improving outcomes for patients living at home with Type 2 Diabetes. Using Bluetooth technology, clinicians can instantly monitor patients’ blood sugar, weight, blood pressure, and heart rate as soon as the patient measures their vital signs. Clinicians can also initiate video visits in which the clinician and patient can discuss patient management of their Type 2 Diabetes.

Recent meta-analyses showed that telehealth interventions result in a modest but significant improvement in reductions in glucose levels when compared to usual care [14, 15]. In addition, two 2016 systematic reviews, one based on 111 randomized controlled trials (RCTs) and another on 55 RCTs, found that home telemonitoring significantly improves glucose management; however, less than 25% of RCTs were conducted with ethnically diverse patients, thereby diminishing the external validity of the findings [16–18] Polisena et al.’s [19] meta-analysis of 26 studies revealed a positive effect on glucose management, noting that more research of higher methodological quality is required, recommending the inclusion of patients from diverse backgrounds to increase external validity and assess technology adaptation to optimize use among different populations [19]. Other meta-analyses on underserved populations, which conclude that home telemonitoring improves glucose management, note that studies should utilize community-based participatory research (CBPR) approaches involving patients and caregivers to develop personalized interventions to enhance persistence in usage and treatment adherence [20–22]. Similarly, a 2017 overview of systematic reviews of mHealth Type 2 Diabetes interventions identified significant reductions in HbA1c when compared to comprehensive outpatient management (COM) [23]. One meta-analysis of randomized trials using home telemonitoring showed improvements in Type 2 Diabetes patient problem areas [18]. Greater well-being was also reported by patients using home telemonitoring compared to usual care [20].

Few studies, however, have examined telehealth technologies in patients from underserved populations or have specifically tailored them to fit the cultural, health literacy, and other needs of underserved groups [24]. The IDEATel study demonstrated that home telemonitoring technology improved underserved patients’ blood pressure, cholesterol, and HbA1c, but the study did not focus exclusively on H/L populations [25]. While IDEATel’s seminal results are encouraging, iterative tailoring based on stakeholder input is required to optimize outcomes specifically for underserved and understudied H/L populations.

Home telemonitoring programs have shown promise in improving glucose management in underserved populations [25, 26]. The American Diabetes Association recommends patient-centered management strategies for Type 2 Diabetes [27], but there has been no effort to date to specifically tailor a comprehensive, evidence-based home telemonitoring program to meet the needs of patients from H/L underserved populations. Interventions based on models that involve cultural, personal, caregiver, and community factors and tailor care to patient preferences have demonstrated greater effectiveness [28]. Our research is germane to the National Academy of Medicine’s priority topics for comparative effectiveness research, which recommends comparing home telemonitoring and COM in managing chronic disease and enhancing medication adherence [29]. Although effective in the general population, it is unclear how home telemonitoring should be tailored to meet the needs of H/L underserved patients. Our study seeks to fill critical knowledge gaps identified by meta-analyses, systematic reviews, and the National Academy of Medicine by directly comparing home telemonitoring to COM, an existing best practice based on 2018 American Diabetes Association Standards of Medical Care in Diabetes [30].

Culturally tailored interventions may improve patient satisfaction, program adherence, and, ultimately, clinical outcomes [12]. The discrepancy in diabetes disease burden between racial, ethnic, and income groups necessitates that researchers tailor effective interventions for acceptability and relevance for those in populations at greatest risk, such as H/L populations. One approach, CBPR, is a collaborative partnership between key stakeholders from differing backgrounds and perspectives and researchers to address the gap between science and “real world” practice through joint decision-making. Stakeholders include patients, caregivers, providers, community-based organizations, public health officials, diabetes organizations, disparities experts, as well as health policy and payer representatives, and other persons of interest [31]. These stakeholders are asked to guide the study team concerning decisions such as: defining the research question, the collection and analysis of data, interpretation of findings, and dissemination of results [32].

Using a CBPR approach in formative research facilitates accurate tailoring and increases the likelihood of acceptability and successful replication [33]. CBPR involves recognizing community members as “equal partners” in the conduct of research, to adapt interventions so they are acceptable and effective in target communities in a replicable manner [32, 34]. CBPR has been used to adapt programs in a variety of areas (including home telemonitoring in heart failure [34], COPD tele-exercise [35], mental health [36], cancer [37], sexually transmitted infections [38–40], and smoking [41]). However, there has been a paucity of literature regarding home telemonitoring adaptation in patients with Type 2 Diabetes from underserved communities [20–22].

This research endeavors to culturally adapt a home telemonitoring program for H/L patients with Type 2 Diabetes from underserved communities in the New York Metropolitan area and formally test it in an RCT to assess whether home telemonitoring is effective in improving outcomes for H/L patients with Type 2 Diabetes. This paper describes the application of CBPR mixed methods to culturally tailor and adapt a home telemonitoring program for H/L patients with Type 2 Diabetes, using feedback from key community and stakeholders to optimize the intervention for this target population.

## Methods

### Design

The CBPR approach used to adapt the home telemonitoring program for H/L patients with Type 2 Diabetes herein utilized the structured ADAPT-ITT [39] model for modifying evidence-based interventions. The ADAPT-ITT model, a pragmatic framework that utilizes iterative, experiential processes in the form of eight sequential phases, was used to guide the adaptation of the intervention. As highlighted below, the ADAPT-ITT framework incorporates stakeholder input in all phases of the model (see Table 1 for a summary of procedures completed).

**Table 1:** Completed ADAPT-ITT procedures [39]

ADAPT-ITT phase	Methodology
1. Assessment	Conducted focus groups/needs assessment with Community Advisory Board and subcommittees
2. Decision	Decision regarding type of intervention was pre-determined by evidence base; however, decisions regarding major adaptations to the intervention were implemented by the study team based upon Community Advisory Board recommendations (equipment and study structure)
3. Administration	Theater testing was conducted during the Community Advisory Board patient panel focus groups with patient stakeholders iteratively (3 times across 8 months) prior to intervention implementation.
4. Production	A draft of the tailored intervention was iteratively (3 times across 8 months) presented to the Community Advisory Board for further feedback and approval.
5. Topical experts	The study team specifically recruited topical experts for Community Advisory Board membership; see Fig. 1.
6. Integration	Community Advisory Board input was integrated into the final adapted version of the intervention; the telehealth software company revised the software to reflect Community Advisory Board recommendations.
7. Training	Both clinical and recruitment specialists received training with regard to study structure and equipment use.
8. Testing	A pilot test was conducted with 12 patients to identify “hands-on” challenges requiring adaptation; a focus group and structured interviews were subsequently held with these patients to further explore these challenges and solutions

**Fig. 1:**
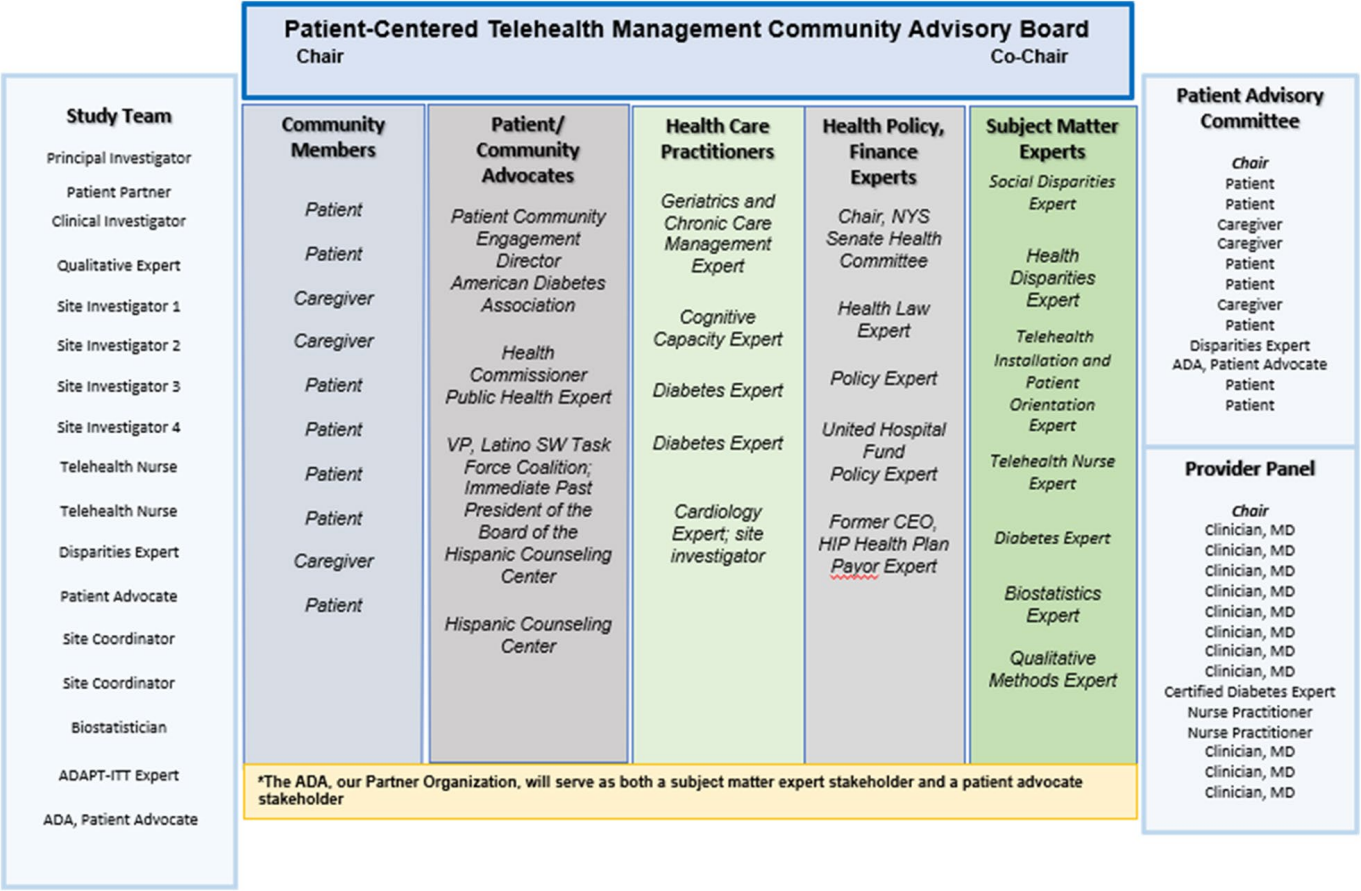
Community Advisory Board Membership

## ADAPT-ITT phases

### Phase 1 (assessment)

The first phase to adapt the home telemonitoring intervention entailed the establishment of a community advisory board (Community Advisory Board, Table 2). Community Advisory Board members (*n* = 23), comprised of key stakeholders, included: H/L patients with Type 2 Diabetes and nonprofessional caregivers; disparity experts, clinicians (geriatricians, endocrinologists, primary care physicians, and nurses), patient advocates, and payor and health policy representatives. These individuals advised the study team on all aspects of study design, implementation, and evaluation. More specifically, the Community Advisory Board was responsible for program tailoring, identifying factors adversely impacting acceptance/feasibility among this population, and reducing the impact of such factors on usability. Both Community Advisory Board members and patient stakeholders were compensated $50 for participation. A qualitative consultant led the focus group discussions with content guided by predetermined topics outlined in an interview guide including instructions to prioritize patient stakeholder contributions above medical and professional stakeholders [42]. All focus groups and structured interviews were conducted in a private conference room and recorded with written patient consent. Phase 1 took place during the first 8 months of the study (January–August 2019).

**Table 2:** Study adaption processes

Methods	Process
Community Advisory Board Patient Advisory Committee (Spanish) Provider Advisory Committee (English)	Focus groups of Community Advisory Board Focus groups of Patient Advisory Committee Focus groups of Provider Advisory Committee January 2019 March 2019 September 2019
Pilot	June/July 2019 Patient focus group for completed HTM – August 2019 Patient interviews for HTM dropouts – August 2019 Patient interviews for completed COM – August 2019 Patient interviews for COM dropouts – August 2019
Extended Study Team	Biweekly meetings In-depth discussion of challenges and recommendations of Community Advisory Board and Pilot data

In addition to the full Community Advisory Board, a patient advisory committee was formed to limit the influence and possible intimidation of patients and community members by providers and policymakers. The patient advisory committee sessions were conducted in Spanish. Similarly, a provider panel met separately to review the intervention and study approaches, and to facilitate acceptability to the provider community. The provider panel sessions were conducted in English. Community Advisory Board subcommittee meetings were held directly after full Community Advisory Board meetings.

### Phase 2 (decision)

Phase 2 involved the review, selection, and decision to adopt or adapt an intervention. The selection of the intervention itself (see description of intervention below) was pre-determined by grant funding from the Patient Centered Outcomes Research Institute. As such, this was the only phase that could not be completed in its entirety. Phase 2 took place during the first 8 months of the study (January–August 2019).

### Phase 3 (adaptation)

In this phase, we utilized theatre testing of the intervention. First, the study staff demonstrated the telemonitoring equipment to the Community Advisory Board, where the clinician contacted the “patient” (represented by study staff) on the tablet. This demonstration included the use of peripheral devices (Bluetooth-enabled glucometer, weight scale, and blood pressure cuff), educational videos, and patient data capture screens.

Immediately after the general demonstration, patients were divided into Spanish-speaking and English-speaking groups and proceeded with theater testing, wherein the tablets were given to patients to use and critique using the “think aloud” strategy. Patients also were asked to view and discuss several representative pre-selected educational videos to review. Likewise, feedback was simultaneously obtained by the provider panel. Provider subcommittees tested the English screens and were conducted in English. Each screen of the intervention was thereby “used” and “reacted to” in terms of intervention adaptation.

Although feedback from all Community Advisory Board members was incorporated, the study team gave particular weight to the patient advisory committee feedback. This phase was repeated twice (February/March and August/September 2019), to ensure that every adaptation met the spirit of the committee’s recommendations (see Table 2).

### Phase 4 (production)

Phase 4 resulted in the production of a first draft of the adapted intervention. This first draft was presented at a second Community Advisory Board meeting in March 2019.

### Phase 5 (identification of topical experts)

During Phase 5, content area experts were identified to provide expertise to the process. In the current study, the research team identified key stakeholders at project inception; in addition, the Community Advisory Board identified additional patients, caregivers, and community-based organizations to join the Community Advisory Board (January 2019).

### Phase 6 (integration)

Phase 6 involved the formation of a more “finely tuned” draft of the intervention based on input from topical experts. Thus, a second draft, which was based upon feedback (obtained June–July 2019) from patients participating in the pilot study during April–June 2019, was presented at a third Community Advisory Board meeting in September 2019.

As can be seen in Table 3, focus groups (*n* = 3) and structured interviews (*n* = 12) were also conducted with pilot study patients directly after the four-month pilot study, both with patients who dropped out of either arm of the pilot study as well as those who were randomized to the COM study arm to identify ‘on the ground” barriers. Results of the focus group conducted in the first few months of the adaptation process were used to develop questions asked in the structured interviews with pilot study patients.

**Table 3:** Detail of study component administration

Month	Study component
January	Community Advisory Board Focus Group: Confirm Outcomes, Preliminary Intervention Adaptions & Study Design
	Patient Advisory Board Focus Group: Confirm Outcomes, Preliminary Intervention & Study Design Adaption Recommendations
	Provider Focus Group Pre-Pilot Adaptation Recommendations
February	Community Advisory Board, Patient Advisory Board, Provider Focus Group Analysis
	Pre-Pilot Adaptation Approval by the Community Advisory Board
	Pre-Pilot Adaptation Complete
March	Pilot
April	
May	
June	
July	Pilot Patient/Caregiver Focus Groups
	Pilot Analysis
August	Patient Advisory Board Focus Group: Post-Pilot Adaptions
	Provider Focus Group: Post-Pilot Adaptions
	Community Advisory Board Finalizes Intervention Adaption

Based on pilot patient feedback, two major adaptations were implemented. First, we identified alternate scales to decrease the total number of questions the participant had to answer while still capturing the desired constructs via validated instruments. Second, we recommended a modification of our protocol to the study funder from a traditional RCT to a randomized consent design approach with the Zelen method [43], which was ultimately approved and implemented. Specifically, the Zelen approach involved the use of two separate consent forms: 1) acquiring a subject’s permission to follow their data over time; and, 2) consenting the patient to telemonitoring [ 43].

### Phase 7 (training)

During Phase 7, the staff was trained on the implementation of the updated version of the intervention. Specifically, the recruiters were trained in scale administration as well as specific areas of changes to study structure (e.g., how to approach patients with different consent forms once the Zelen method was implemented as a randomization approach). Phase 7 took place in June and July 2019.

### Phase 8 (testing)

Finally, Phase 8 encompassed pilot testing of the most current version of the intervention with “live patients” in the home. Focus groups and structured interviews were conducted with 12 patients enrolled in a 1-month pilot study, to obtain feedback from patients in the home and identify additional barriers or challenges to implementation in the actual home setting. Phase 8 took place in August 2019.

### Participants and population

Pilot study participants (*n* = 12) were self-identified as Latin-X/Hispanic patients with Type 2 Diabetes receiving care from outpatient clinics in the New York Metropolitan area**.**

## Qualitative data analysis

All focus group discussions and structured interviews were audio-recorded and professionally translated and transcribed. The focus groups were approximately 2 hours. Structured interviews were approximately one-half hour. Structural coding was used to mark responses to topical questions in the interview guide [44]. Following a review of the a priori topics, the facilitator developed a codebook to categorize the data and identify salient themes and relationships [45, 46]. The main themes that emerged from the text identified specific recommendations for intervention adaptations.

## Description of the home telemonitoring intervention

HTM is a monitoring system that connects the patient from their home to a provider station via a tablet that the patient uses to communicate over the six-month study period. Home telemonitoring has three main components: 1) provides the patient with basic daily vital signs monitoring and facilitates nurse recognition of sugar levels that are outside the recommended range, 2) a weekly telemonitoring face-to-face video chat between the patient and the nurse, and 3) culturally congruent educational videos that are meant to provide the patient with advice and guidelines in managing their condition. The home telemonitoring system utilized in the present study was provided by Health Recovery Solutions (HRS) Remote Patient Monitoring Platform and was available in both English and Spanish.

### Component 1: daily vital signs

Patients are trained to utilize the tablet and peripherals by the clinician and recruitment specialist. Daily transmissions of Bluetooth-enabled measures included: glucose control (blood sugar as measured by glucometer), blood pressure, and weight, pulse/heart rate. In addition, patients can manually record medication adherence and physical activity. Patient data are stored on a secure, encrypted database and reviewed daily by the study nurse to ensure that patient vital sign values are not outside of recommended targets, as defined by the physician.

### Component 2: telemonitoring visit

Once a week, patients are asked to connect for a weekly scheduled telehealth visit with a nurse. Through this weekly “virtual” visit, clinicians review a patient’s vital signs and discuss the prior week’s data concerning medications; exercise, and nutrition to further involve the patient in his/her care.

### Component 3: educational videos

Weekly, patients are asked to view different educational videos by the nurse. The videos address important diabetes management topics such as: How to Create a Healthy Plate/Cómo Crear un Plato Saludable; What is Type 2 Diabetes?/¿Qué es la Diabetes Tipo 2?; How to Test your Blood Sugar/Cómo Hacerse la Prueba de su Nivel de Azúcar en la Sangre; How do Diabetes Medications Work in the Body/¿Cómo Funcionan los Medicamentos para la Diabetes; Enjoy Exercise with Diabetes/Goce el Ejercicio con Diabetes; Family Fun Brings us Together/La Diversión en Familia une a la Familia; How Can you Succeed with Diabetes/Triunfe con Diabetes; Insulin Keeps you Healthy/La Insulina lo Mantiene Sano. These videos were licensed for use by this study by Kaiser Permanente Medical Group (KPMG).

## Results

Adaptations to the intervention varied widely: from **technology acceptance and consent process concerns** to screen/verbiage (in English and Spanish) changes, desire for additional tablet training, and rejection/reselection of educational videos and hours of operation. The sections below describe the general themes and subthemes that emerged from qualitative analyses of 1) the Community Advisory Board and stakeholder committee (patient and provider panel) focus groups, and 2) the focus groups and interviews from the patient pilot test. Additionally, adaptations made to the intervention are discussed.

### Community advisory board and stakeholder focus groups

#### Technology acceptance concerns

During the community needs assessment, a major theme that quickly emerged from clinician stakeholders centered around technology acceptance by the patients. Several clinicians noted that patients would be hesitant to accept the devices into their homes attributable, in large part, to fear that patients and their families could be “watched.” Additionally, clinicians opined that patients in the study population were likely to require help from their extended families to use the technology. These sentiments were echoed in the patient panel, where the importance of having to train patients on using the technology was highlighted.

*“But for people that come from a place where the technology didn’t exist, it may be shiny and sparkly, but you have to assume if this is going to be successful that you’re starting from a position where you have to train people from the most basic components of it if we’re going to be successful.”*

*“The training has to be so basic and so engaging that they don’t feel intimidated by the actual – it’s like when people have a microphone and they never talk. Hey, does this thing work? It’s the same thing.”*

Other themes that emerged during the community needs assessment included the importance of access to the nurse, flexibility in scheduling appointments, and using clear policies during recruitment. Furthermore, changes to the intervention tablets included requests for lighter tablets, larger font sizes, a log on or blackout screen for added security, and the need for culturally sensitive components such as language and culturally appropriate videos and food choices.

#### Tablet interface feedback

During the theater testing of the intervention, three major themes emerged regarding the tablet interface: 1) presentation of the information; 2) language use; and 3) irrelevant information. Patients reported preferences for how the screen should look to best convey the information. Four individuals noted the use of pictures would be more appropriate, given that a significant portion of the study population may be illiterate.

*“We could have icons! We have different icons of people doing…”*

*“Because if we do image and text, I think that would be much more beneficial.”*

Additionally, four patients noted that the information displayed was overwhelming and that too much information was being presented per screen.

*“I feel like it has too many options.”*

*“I think we need a clearer dashboard…part of the confusion is that those things, people are not understanding what they are.”*

*“I think the point is that it’s very busy.”*

Finally, participants requested the inclusion of additional examples, particularly regarding what is considered exercise.

*“When you’re talking about exercise, I’m thinking about going on a treadmill or maybe I do the garden. And that is considered to be an activity as perfect as to do the treadmill.”*

*“So, I just think that as long as people know that all of those things [exercise] count, that’s what’s important.”*

When reviewing the language used on the tablets, patients noted changes in terminology that should be made to make the instructions more understandable to the study population. It was suggested that language used on the tablets and videos should be at a fourth-grade comprehension level or less and assume a similar level of health literacy.

*“A lot of Spanish people they, they use “sugar” [levels] rather than “glucose.””*

*“So, I don’t know if ‘press’ versus ‘touch’ is the right word….or ‘verify the oxygen level’.*

*“I’m assuming that’s not a fourth-grade level either for sure.”*

Clinicians also identified several instances in which information included on the screens was irrelevant to the current study, thus risking overwhelming the patients. These included requiring patients to measure their temperature daily as well as recording oxygen saturation.

In the final Community Advisory Board focus group, additional changes to the tablet were discussed. Three subthemes were identified: 1) screen or verbiage changes; 2) desire for more training on using the tablet; and 3) video feedback.

Participants noted that translations, despite being adapted once, still needed further refinement. This articulates one of the hallmarks of adaption, the process needs to be iterative and responsive to recommended modifications.

*“I understand it but I couldn’t tell you what these translations are.”*

Participants also expressed a desire for more training and/or explanation on tablet use. As previously discussed, the study population required substantial assistance from a caregiver to effectively use the technology, thus more information on using the device was requested.

*“In other words, or for example, you know, uh, you’re going to hear a sound. I don’t know exactly how it works. But, you know, or you’re going to feel it pressuring, whatever it is that they’re going to be feeling so that they are part of the process.”*

New videos developed by KPMG were presented to the Spanish-speaking patient groups, which were considered more acceptable and culturally congruent, both in terms of homophily (the actors “look like me”) and the foods/cultural practices presented. Participants greatly preferred these new videos, while noting additional aspects of the video(s) that were not culturally appropriate or hard to understand for the given population. Participants liked the simplicity of the videos.

*“But I think that’s the only thing is culturally going back to is very often, people measure by spoon. They don’t necessarily use measuring cups, which we will try to teach them, but most people don’t. Most people do it by their spoon.”*

#### Video review

HRS videos were presented to the patient panel (in Spanish) and provider panel (in English) during the first and second Community Advisory Board meetings. Video reviews of the Spanish- and English-speaking groups were analyzed to identify common themes and areas needing adaptation. Five themes emerged for the educational videos: 1) repetition of information, 2) presentation of information, 3) language choice, 4) cultural incongruence, and 5) personal connection with actors.

Participants in the English-speaking group expressed the importance of repetition of information presented throughout the educational videos, in reinforcing and understanding the message being delivered.

*“I thought it was really important that on two different occasions they talked about exercise and physical activity.”*

*“I thought the recap at the end really summed it up nicely.”*

Both groups noted that certain aspects of the educational videos made it difficult to understand the messaging. These challenges included the narrator speaking too fast and the framing of the information being presented. Specifically, while it is important to communicate the dangers associated with uncontrolled diabetes, it is just as important to outline the steps one should take to avoid the danger and foster better health.

*“It’s kind of a downer. Like I get it. I like how they were honest about their feelings. But not until the last sentence of so what do you do about it. They could have spent more time with that.”*

*“No, it scared me at the very beginning. I mean, imagine we’re already scared when we hear about the diagnosis of diabetes and now you’re telling me all these frickin’ complications at the very beginning. It’s like just shut off light.”*

Both groups also expressed concerns regarding the complicated verbiage used throughout the videos. These concerns included suggestions for changing more complex verbiage to simpler words that would be easily understood, including definitions for words, and being provided additional educational information.

*“Ophthalmologist and podiatrist, that’s a little bit complex. They should have said eye doctor or foot doctor just to simplify.”*

*“I had to look up what urinating meant. I didn't know what urinating meant.”*

*“…the word statistic is probably not even a very familiar word for a lot of people.”*

Spanish-speaking participants emphasized the need for the videos to be tailored to be more culturally appropriate. Participants highlighted issues with the translation in the videos being inaccurate as well as the food being presented being representative of a traditional American, rather than a Hispanic, diet.

*“It needs to be culturally appropriate for Spanish folks. So, there was nothing about this that made me feel as a Spanish speaker, I should be watching this video versus any other – it seems like it was an English translation into Spanish as opposed to a transcultural…”*

*“You also – it says Latino population. It didn’t seem to be food from the Caribbean or South America. It looked like an American diet.”*

Spanish-speaking participants also noted a need for a personal connection with the video actors for the information to be most effective. Most common among these concerns was an inability to relate to the actors (e.g., “someone like me” i.e., homophily), who were American actors with dubbed over Spanish voice.

*“There was no connection and that’s a big problem with my mother…”*

*“It didn’t have any cultural connection, like for my mom…”*

The committees requested that the study team identify alternate, culturally congruent videos that more aligned with the cultural experiences and needs of the target population. The study team searched for educational videos that met the requirements of the committees. New videos that were developed by KPMG were presented at the third Community Advisory Board meeting and found to be acceptable by the committees. Thus, these videos were incorporated into the final intervention (without KPMG’s logo).

#### Consent process concerns

In the final Community Advisory Board focus group, study procedures were reviewed, and concerns about the consent process emerged. Individuals raised concerns regarding the consent process and how potential study participants were being approached. The process was perceived as overwhelming in terms of the amount of information being presented and the length of the consenting process. These issues were further compounded by the timing in which the recruiter was approaching patients.

*“At that point, they have already seen the doctor, they’ve asked their questions and they’re checking out. They’re, they’re -- want to do is make their next appointment that they might not be as interested in hearing from [recruiter].”*

### Feedback from pilot study participants

Information gathered from a focus group and individual interviews with participants in the pilot study, suggest strong acceptability despite concerns around the consent process and that their private health information would remain confidential using the tablets. Participants expressed positive sentiment regarding the consent process and its ease of completion. Participants also were pleased with the level of information provided regarding the study, although the time required for both the consenting process and survey completion was reported to be onerous.

*“Everything was very easy. [The recruiter] explained everything and that made the process even easier.”*

*“I wanted to hear a verbal explanation of what I was going to do and how it was going to help me. And that is why it took longer.”*

Participants expressed a desire to enter the program because it targeted Latino/Hispanic people specifically. Participants also yearned to learn more about diabetes and how to manage the disease, in general.

*“Honestly, sometimes as a Hispanic you tend to doubt this type of help…[the recruiter] explained the program to me and I am very interested because I have seen the consequences of diabetes.”*

Participants involved their immediate family as caregivers throughout the study.

*“I think that the right person, in my opinion, is a relative.”*

*“My family also got very involved and they were looking out for my weight and the food I ate. All of that is very important.”*

Participants considered the Spanish speaking nurse to be integral to the study, and that the trust developed between patient and nurse was crucial. Additionally, the scheduling flexibility afforded to participants for virtual visits was important.

*“Yes, the most important thing for me was the language because my doctor speaks English and I was more comfortable speaking to [the nurse] than with my doctor.”*

*“I got home late and she called me and called me and when I got home, I called her and told her, we should do this tomorrow because it’s too late. And she said, [name], it’s fine. And I was sorry because I explained to her that just like him, I come home from work very late. But she – she worked around my schedule.”*

Finally, participants were skeptical of the tablet and felt their privacy may be compromised with the device in their home.

*“He felt suspicious because the tablet was there all the time and he felt like he was being watched.”*

*“It would be good if the personnel could tell us this before they hand us the tablet. Tell us that will not be a problem.”*

*“They thought they could spy us through the camera.”*

### Adaptations implemented as a result of stakeholder feedback

**Fig. 2:**
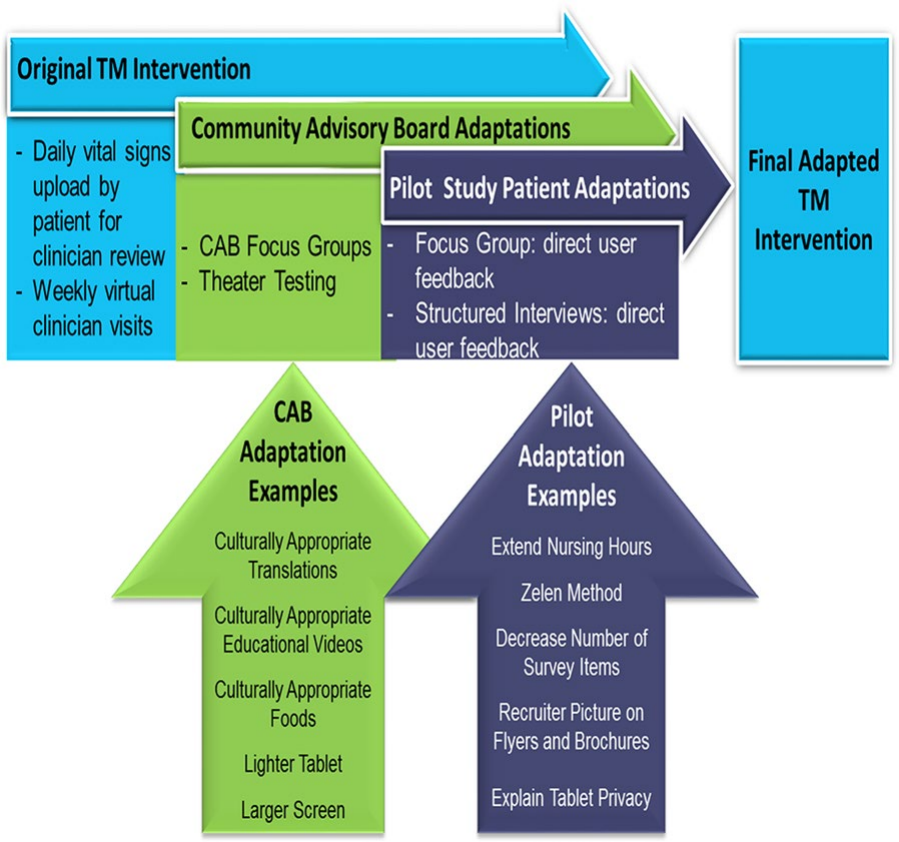
Adaptations Implemented as a Result of Stakeholder Feedback

A significant number of adaptations were made to the original home telemonitoring program to adapt it to the needs of the underserved Hispanic population with Type 2 Diabetes. As can be seen in Fig. 2, in general, these changes were in two forms: 1) changes to the patient-facing screens on the tablets themselves, and 2) changes to the study design/enrollment procedures.

#### Changes to the patient-facing tablet screens

When presenting each patient-facing screen and educational video to stakeholders on the Community Advisory Board and its subcommittees, many adaptations were recommended. First, culturally appropriate translations (e.g., using the word “azucar” rather than “glucosa”) system were incorporated into the system. Second, diabetes educational videos that were first presented as part of the HRS system were substituted with the KPMG educational videos, which were considered to be more culturally congruent, and not just “English folks eating American foods dubbed over in Spanish”. Third, patients asked for lighter tablets that were more portable for use at work, with privacy/logon screens. Finally, larger screens and font sizes were requested to accommodate older, diabetic eyes.

#### Changes to study procedures

In addition to the Community Advisory Board and subcommittee feedback, when conducting focus groups and structured interviews with patients who had participated in a one-month pilot program, several study procedure adaptations were identified. First, a request was made to extend nursing telemonitoring hours to include nights and weekends, as a significant proportion of the patient population was working during the 9–5, Monday-Friday workday. A second recommendation, arising from pilot patient feedback referencing an extremely lengthy consent process, resulted in modifying our protocol, from a traditional RCT to a randomized consent design. This approach is particularly useful in lifestyle interventions, which are often complex and subject to important factors, such as patient preference and non-adherence. A third adaptation, also arising from pilot patient feedback, resulted in a decrease of survey items, which patients considered to be onerous, taking over an hour and ½ to administer. Fourth, patients recommended that a picture of the recruiter be included on recruitment brochures at the clinics, so that patients would recognize the “stranger” that was approaching them for study participation. Finally, there was some fear expressed by patients that: 1) their information could be shared with federal agencies like the INS to identify them, or that the tablets could “listen to” subjects when they were not using them to interact with the nurse. This resulted in the inclusion of an extensive explanation of their privacy protections as part of the enrollment process, including the execution of Certificate of Confidentiality by the National Institutes of Health, which protects the privacy of research subjects by prohibiting disclosure of identifiable, sensitive research information to anyone not connected to the research.

## Discussion

While previous meta-analyses and systematic reviews have documented the clinical efficacy of remote monitoring of patients with diabetes and emphasized the importance of adapting interventions to facilitate cultural relevance, few efforts have been made to adapt telemonitoring interventions [40, 41, 47, 48]. The present study utilized a formative process to CBPR, namely ADAPT-ITT, to optimize home telemonitoring utilization among H/L patients with Type 2 Diabetes from underserved communities. Specifically, focus groups and structured interviews were conducted to gather feedback regarding the intervention which was subsequently used to make appropriate adaptations to the intervention.

Two major themes emerged from the qualitative data: technology acceptance concerns and consent process concerns. Our findings echo previous work that adapted telemonitoring interventions in underserved communities for COPD and heart failure telemonitoring [35]. These themes appear to be overarching constructs that exist regardless of disease, suggesting that it is important to address these concerns when targeting underserved populations. For example, proving relevant food selections (the Caribbean or South American diet versus the American diet), and utilizing the right terminology (azucar rather than glucose) supersedes disease category.

The same is true for our second theme: changes to study procedures. Patients across studies discussed being able to change program hours of operation to adjust for work schedules. In the present study, night and weekend televisits were added to weekday visits to offer flexibility for those patients working full time.

### Strengths and limitations

There are several strengths of this study. The use of an adaptation framework to systematically adapt the home telemonitoring intervention ensures a comprehensive approach that addresses many aspects and perspectives that might be otherwise be missed. For example, the eight steps presented in Table 1 ensures a comprehensive community assessment, qualitative capture of important stakeholder perspectives, repetitive theatre testing during adaptation phases, continuous Community Advisory Board feedback and approval of the intervention, the input of topical experts, training of both clinical and recruitment specialists, and patient pilot testing with subsequent focus group and structured interviews to facilitate the identification of “hands-on” challenges that might not be readily anticipated by the Community Advisory Board alone.

Study limitations include a relatively small sampling (5%) of pilot patient participation in the focus group. Based on anecdotal evidence, we believe that while this 5% accurately represented the concerns of the current patient population, it may not represent concerns of patients in other settings (i.e., rural areas). Most suggested changes to both study structure and equipment were implemented, although there were a few suggestions that could not be achieved, due to resource limitations.

### Future research

Given the significant number and types of adaptations implemented in this study (both as a result of stakeholder feedback and pilot study participant feedback), it is a reasonable assumption that future home telemonitoring interventions incorporate systematic adaptation methods such as ADAPT-ITT to ensure usability in target populations. It is indeed unfortunate that usability testing, used as a standard approach in product marketing (i.e., testing an interface to ensure easy navigation, thereby increasing the likelihood that the user is going to stay on that website), is often so lacking in intervention development in health care.

## Conclusions

There has been a dearth of literature regarding adapting telehealth interventions for H/L patients with Type 2 Diabetes from underserved communities. This qualitative study identified adaptations that are important to ensure that a complex intervention is generalizable for patients from underserved H/L communities. Specifically, this study emphasizes the applicability of Wingood and DiClemente’s ADAPT-ITT framework as a tool in systematically adapting a home telemonitoring intervention for patients with Type 2 Diabetes from underserved H/L communities [39]. Although the results of the randomized trial will not be available for several months, our findings from this initial qualitative phase demonstrate that using ADAPT-ITT, a remote home monitoring intervention can be adapted to reach patients who are most likely to experience access issues, through the input of the patient population as well as other important stakeholders.

## Data Availability

Qualitative data, including transcriptions of audio recordings, was the only type of data used for this study. As we have assured focus group participants that their study records would remain private, it is not possible to make the data publicly available.

## Abbreviations

H/L: Hispanic/Latino
RCT: Randomized controlled trials
CBPR: Community-based participatory research
COM: Comprehensive outpatient management
HRS: Health Recovery Solutions
KPMG: Kaiser Permanente Medical Group
